# One-Step Biosynthesis of Vitamin C in *Saccharomyces cerevisiae*

**DOI:** 10.3389/fmicb.2021.643472

**Published:** 2021-02-25

**Authors:** Mengyu Zhou, Yanhui Bi, Mingzhu Ding, Yingjin Yuan

**Affiliations:** ^1^Key Laboratory of Systems Bioengineering (Ministry of Education), Frontier Science Center for Synthetic Biology, School of Chemical Engineering and Technology, Tianjin University, Tianjin, China; ^2^Collaborative Innovation Center of Chemical Science and Engineering (Tianjin), Tianjin University, Tianjin, China

**Keywords:** vitamin C, *Saccharomyces cerevisiae*, protein engineering, copy number engineering, synthetic biology

## Abstract

Vitamin C (VC) is comprehensively applied in foods, cosmetics, pharmaceuticals, and especially clinical medicine. Nowadays, the industrial production of VC mainly relies on the classic two-step fermentation route, and researchers have explored the way for one-step fermentation of VC in recent years. In this study, a VC biosynthesis pathway that directly produced VC from glucose was reconstructed in *Saccharomyces cerevisiae*, and the protein engineering and metabolic engineering strategies were adopted to improve it. First, five exogenous modules from *Arabidopsis* were introduced into the chassis cells by synthetic biology approaches to obtain the strain YLAA harboring VC biosynthesis. In addition, L-galactose dehydrogenase (L-GalDH) and L-galactono-1,4-lactone dehydrogenase (L-GLDH) were fused and expressed in *S. cerevisiae* cells for the first time, which increased the intracellular VC accumulation by 2.78-fold, reaching 9.97 ± 0.09 mg/L. Through copy number engineering, it was further confirmed that the last step catalyzed by L-GLDH is the rate-limiting step. GDP-L-galactose phosphorylase (GPP) encoded by vtc2 is another rate-limiting enzyme confirmed by GAL1p overexpression results. Finally, by balancing gene expression and cell growth, the highest production strain with overexpressing vtc2 by multicopy plasmids was constructed. The VC accumulation reached 24.94 ± 1.16 mg/L, which was currently the highest production from glucose in *S. cerevisiae*. The production of the recombinant strain reached nearly 44 mg/L with the exogenous addition of L-galactose or glutathione. The results further emphasized the importance of the step catalyzed by GPP. The investigation provided experience for the efficient biosynthesis of VC and the determination of rate-limiting steps.

## Introduction

Vitamin C (VC) is a water-soluble vitamin, which is closely related to human beings. For more than 80 years since the discovery of VC, its physiological activities and biological functions have been continuously developed (King and Waugh, 1932; Naidu, 2003; Grosso et al., 2013; Camarena and Wang, 2016). As a result of the strong reducing ability of ascorbic acid, it is an important antioxidant, free radical scavenger, and one of the nutrients for preventing sepsis (Padh, 1990; Timoshnikov et al., 2020). By the 1950s, VC was considered to have an anti-cancer effect by participating in the redox reaction to affect the growth of cancer cells and even kill them (McCormick, 1954; Yun et al., 2015; De Francesco et al., 2017). Recently, researchers discovered VC advanced plasma cell difference to enhance antibody production, and high injecting doses enhanced the effectiveness of antibodies and slowed down or even prevented the growth of cancer (Magrì et al., 2020; Qi et al., 2020). What is more, VC could also be used in the treatment of leukemia by regulating the number and function of blood-forming blood stem cells and reducing the patients’ pain as a cofactor for the synthesis of substances that had analgesic effects (Prigge et al., 2000; Yeom et al., 2007; Harrison and May, 2009; Agathocleous et al., 2017; Carr and McCall, 2017; Cimmino et al., 2017).

Nowadays, the industrial production of VC mainly relies on the classic two-step fermentation method (CTFR) to convert D-sorbitol to 2-keto-L-gulonic acid (2-KLG, the precursor for VC) (Reichstein and Grüssner, 1934; Pan et al., 2017). Afterward, researchers improved the fermentation process to optimize CTFR by the improvement of the fermentation process and the reconstruction of microorganisms, such as the exogenous addition of cofactors or specific complex nutrients (Gao and Yuan, 2011; Zou et al., 2012; Huang et al., 2013), the reconstruction of the helper microorganisms (Zhou et al., 2011; Du et al., 2012; Zhu et al., 2012a, b), the optimization of the metabolic pathway in the production microorganisms (Cai et al., 2012; Du et al., 2013; Pan et al., 2017), and a new design of bacterial consortia (Mandlaa et al., 2013; Jia et al., 2016).

In addition, researchers have been working on the production of VC in one-step fermentation over the years. It seemed to be the most direct method for one-step fermentation producing 2-KLG that CFTR-related dehydrogenases were introduced into a certain microorganism (Gao et al., 2014; Zeng et al., 2020). Furthermore, the direct biosynthesis of VC from D-glucose or D-sorbitol was a practical approach. Although most microalgae had a complete path to synthesize VC, its cultivation cost was much higher than that of microorganisms, which greatly limited its application (Running et al., 2002). Yeast could synthesize D-erythorbic acid (D-EAA), a structural and functional analog of VC (Huh et al., 1998). Compared with the transformation process of VC synthesis in plants, the molecular configuration changes in the last two steps were very similar (Kim et al., 1998). With the continuous exploration, researchers gradually discovered the enzymes lacking in yeast to synthesize VC. Rosa et al. (2013) transferred the VC synthesis pathway of plants to *Kluyveromyces lactis*, but the maturity of its genetic manipulation and the breadth of its application were not as good as *Saccharomyces cerevisiae.* Martani et al. (2013) introduced this pathway into *S. cerevisiae*, giving it the ability to synthesize VC with the production of 0.2 mg/L (Branduardi et al., 2007).

In this study, *S. cerevisiae* cells were selected as the chassis cells to construct the strains capable of biosynthesizing VC. Furthermore, the rate-limiting steps of the VC synthesis pathway in *S. cerevisiae* were investigated. The effect of the fusion protein expression, the promoter optimization, copy number engineering or the addition of intermediate products, and GSH on VC accumulation was revealed. Finally, based on the above research, a *S. cerevisiae* strain with the highest production of VC from glucose by one-step fermentation was obtained. The research process can provide an effective reference for designing a one-step fermentation route to efficiently produce VC or other chemicals in *S. cerevisiae* strains.

## Materials and Methods

### Gene Amplification and Plasmid Construction

Plasmids used in this study are all listed in Table 1. Genes synthesized in this study are all listed in Supplementary Table S3. All syngeneic genes for the reconstruction of the VC synthesis pathway in yeasts were optimized with yeast codons and obtained by Kingsley Corporation. The fragments of constitutive promoters and terminators were amplified from *S. cerevisiae* genome. The fragments used for strain constructions and the primers used for fragments for this study are listed in Supplementary Table S1. All fragments were amplified using Phanta^®^ Super-Fidelity DNA Polymerase (Vazyme Biotech.) and were gel purified using a kit (TIANGEN) before cloning. Fragment assembly was performed using the Gibson method (Gibson, 2011) or yeast assembly (Gibson et al., 2008).

**TABLE 1:** List of plasmids used in this study.

Plasmid	Description	Source
p2-4-6-1	pRS416-P_PGK__1p_-gme-HXT7t-P_GPM__1p_-vtc2-CYC1t-P_TDH__3p_-vtc4-GPM1t-P_TPI__1p_-galdh-PGK1t	This study
pGLDH-1	pRS415-P_TEF__2p_-gldh-ENO2t	This study
p2-4-6-1-2	pRS426-P_PGK__1p_-gme-HXT7t-P_GPM__1p_-vtc2-CYC1t-P_TDH__3p_-vtc4-GPM1t-P_TEF__2p_-gldh-ENO2t-P_TPI__1p_-galdh-PGK1t	This study
pGME-VTC2	pRS426-P_GPM__1p_-vtc2-gme-CYC1t-P_TDH__3p_-vtc4-GPM1t-P_TEF__2p_-gldh-ENO2t-P_TPI__1p_-galdh-PGK1t	This study
pVTC2-VTC4	pRS426-P_PGK__1p_-gme-HXT7t-P_GPM__1p_-vtc2-vtc4-GPM1t-gldh-ENO2t-P_TPI__1p_-galdh-PGK1t	This study
pGalDH-GLDH	pRS426-P_PGK__1p_-gme-HXT7t-P_GPM__1p_-vtc2-CYC1t-P_TDH__3p_-vtc4-GPM1t-P_TPI__1p_-galdh-gldh-ENO2t	This study
pVTC2-GLDH	pRS425-P_TEF__1p_-vtc2-CYC1t-P_TDH__3p_-gldh-ENO2t	This study
pALO1-VTC2	pRS425-P_GPM__1p_-alo1-HXT7t-P_TEF__1p_-vtc2-CYC1t	This study
pALO1-GLDH	pRS425-P_GPM__1p_-alo1-HXT7t-P_TDH__3p_-gldh-ENO2t	This study
pALO1	pPRS423-P_TDH__3p_-alo1-HXT7t	This study
pHE-Gal	pRS413-P_GAL__1p_-PGI1t	This study
pGME-Gal	pRS413-P_GAL__1p_-gme-PGI1t	This study
pVTC2-Gal	RS413-P_GAL__1p_-vtc2-PGI1t	This study
pVTC4-Gal	pRS413-P_GAL__1p_-vtc4-PGI1t	This study
pGalDH-Gal	pRS413-P_GAL__1p_-galdh-PGI1t	This study
pGLDH-Gal	pRS413-P_GAL__1p_-gldh-PGI1t	This study
pGLDH-2	pRS413-P_TEF__2p_-gldh-ENO2t	This study
pGME-425	pRS425-P_PGK__1p_-gme-HXT7t	This study
pVTC2-425	pRS425-P_GPM__1p_-vtc2-CYC1t	This study
pVTC4-425	pRS425- P_TDH__3p_-vtc4-GPM1t	This study
pGalDH-425	pRS425- P_TPI__1p_-galdh-PGK1t	This study
pGLDH-425	pRS425-P_TEF__2p_-gldh-ENO2t	This study

As shown in Supplementary Figure S1, five exogenous modules were subcloned into pRS416 (ura3 auxotrophic marker) digested by *Kpn*I and *Not*I, resulting in the p2-4-6-1 vector. The exogenous modules expressing gldh was subcloned into pRS415 (leu2 auxotrophic marker) digested by *Not*I and *Sac*I, resulting in the pGLDH-1 vector.

In order to improve the recombination efficiency, 300 bp homologous fragments were used to construct the plasmids with overexpressing exogenous modules and fused proteins. As shown in Supplementary Figure S2, all exogenous optimized modules were subcloned into pRS426 (ura3 auxotrophic marker) digested by *Kpn*I and *Not*I, resulting in the vectors p2-4-6-1-2, pGME-VTC2, pVTC2-VTC4, and pGalDH-GLDH.

The fragments vtc2-gldh, alo1-vtc2, alo1-gldh, and TDH3p-alo1-ENO2t were obtained according to the designed order by overlap extension PCR (OE-PCR). The first three fragments were, respectively subcloned into pRS425 (leu2 auxotrophic marker) digested by *Xho*I and *Hin*dIII, resulting in the multi-copy plasmids pVTC2-GLDH, pALO1-VTC2, and pALO1-GLDH. The last fragment was subcloned into pRS423 (his3 auxotrophic marker) digested by *Xho*I and *Sal*I, resulting in the multi-copy plasmid overexpressing alo1 (Supplementary Figure S3A).

GAL1p-PGI1t was obtained after OE-PCR, digested by *Hin*dIII and *Not*I and eventually subcloned with linearized pRS413 (his3 auxotrophic marker), resulting in the GAL1p expression cassette. Each exogenous module was, respectively, subcloned into this cassette digested by *Bsa*I, resulting in five vectors pGME-Gal, pVTC2-Gal, pVTC4-Gal, pGalDH-Gal, and pGLDH-Gal (Supplementary Figure S3B). The fragment TEF2p-gldh-ENO2t was subcloned into pRS413 (HIS auxotrophic marker) digested by *Not*I and *Sac*I resulting in the vector pGLDH-2. All fragments of exogenous genes were amplified and then digested by *Not*I and *Sac*I. Each exogenous module was eventually subcloned with linearized pRS425, resulting in five multicopy vectors pGME-425, pVTC2-425, pVTC4-425, pGalDH-425, and pGLDH-425 (Supplementary Figure S3C).

All the restriction enzymes and modification enzymes used in this study were from New England Biolabs.

### Strains, Medium, and Culture Conditions

Yeast strains used in this study are listed in Table 2. Plasmid cloning work and circuit construction characterization were all performed in *Escherichia coli* Trans-T1 strains. They were grown in *Luria–Bertani broth* (LB) media (10 g/L of peptone, 5 g/L of NaCl, and 5 g/L of yeast extract) at 37°C and 220 rpm. *S. cerevisiae* strain BY4741 (MATa his3Δ1 leu2Δ0 met15Δ0 ura3Δ0) was used as the parental strain in this study. All yeast transformations were performed using the LiAc/PEG/ss-DNA protocol. The parental yeast strain was treated under heat shock at 42°C for 15 min, recovered in 5 mM calcium chloride for 5 min after incubation, and eventually plated on a suitable selection medium (Martani et al., 2013). Engineered yeast strains were grown in synthetic complete (SC) medium (20 g/L of glucose, 6.7 g/L of yeast nitrogen base without amino acids, and appropriate amino acids) under auxotroph-screening conditions (Uracil 0.02 g/L, histidine 0.02 g/L, tryptophan 0.02 g/L, and leucine 0.1 g/L) or yeast extract–peptone–dextrose (YPD) medium (20 g/L of glucose, 10 g/L of yeast extract, and 20 g/L of peptone). They were grown in shake tubes at 30°C and 220 rpm.

**TABLE 2:** List of yeast strains and plasmids used in this study.

Strain	Genotype	Description	Source
BY4741	MATa; his3Δ 1; leu2Δ 0; met15Δ 0; ura3Δ 0	/	Our laboratory
YLAA	MATa; his3Δ 1; leu2Δ 0; met15Δ 0; ura3Δ 0	BY4741 (p2-4-6-1, pGLDH-1)	This study
YLAA-2	MATa; his3Δ 1; leu2Δ 0; met15Δ 0; ura3Δ 0	BY4741 (p2-4-6-1-2)	This study
YLAAF-1	MATa; his3Δ 1; leu2Δ 0; met15Δ 0; ura3Δ 0	BY4741 (pGME-VTC2)	This study
YLAAF-2	MATa; his3Δ 1; leu2Δ 0; met15Δ 0; ura3Δ 0	BY4741 (pVTC2-VTC4)	This study
YLAAF-3	MATa; his3Δ 1; leu2Δ 0; met15Δ 0; ura3Δ 0	BY4741 (pGalDH-GLDH)	This study
YLAA*	MATa; his3Δ 1; leu2Δ 0; met15Δ 0; ura3Δ 0	BY4741 (p2-4-6-1, pGLDH-2)	This study
YLAAK-1	MATa; his3Δ 1; leu2Δ 0; met15Δ 0; ura3Δ 0	YLAA* (pVTC2-GLDH)	This study
YLAAK-2	MATa; his3Δ 1; leu2Δ 0; met15Δ 0; ura3Δ 0	YLAA* (pALO1-VTC2)	This study
YLAAK-3	MATa; his3Δ 1; leu2Δ 0; met15Δ 0; ura3Δ 0	YLAA* (pALO1-GLDH)	This study
YLAAK-4	MATa; his3Δ 1; leu2Δ 0; met15Δ 0; ura3Δ 0	BY4741 (p2-4-6-1, pALO1, pVTC2-GLDH)	This study
GME-Gal	MATa; his3Δ 1; leu2Δ 0; met15Δ 0; ura3Δ 0	YLAA (pGME-Gal)	This study
VTC2-Gal	MATa; his3Δ 1; leu2Δ 0; met15Δ 0; ura3Δ 0	YLAA (pVTC2-Gal)	This study
VTC4-Gal	MATa; his3Δ 1; leu2Δ 0; met15Δ 0; ura3Δ 0	YLAA (pVTC4-Gal)	This study
GalDH-Gal	MATa; his3Δ 1; leu2Δ 0; met15Δ 0; ura3Δ 0	YLAA (pGalDH-Gal)	This study
GLDH-Gal	MATa; his3Δ 1; leu2Δ 0; met15Δ 0; ura3Δ 0	YLAA (pGLDH-Gal)	This study
GME-M	MATa; his3Δ 1; leu2Δ 0; met15Δ 0; ura3Δ 0	YLAA* (pGME-425)	This study
VTC2-M	MATa; his3Δ 1; leu2Δ 0; met15Δ 0; ura3Δ 0	YLAA* (pVTC2-425)	This study
VTC4-M	MATa; his3Δ 1; leu2Δ 0; met15Δ 0; ura3Δ 0	YLAA* (pVTC4-425)	This study
GalDH-M	MATa; his3Δ 1; leu2Δ 0; met15Δ 0; ura3Δ 0	YLAA* (pGalDH-425)	This study
GLDH-M	MATa; his3Δ 1; leu2Δ 0; met15Δ 0; ura3Δ 0	YLAA* (pGLDH-425)	This study

Yeast cells for intracellular VC determination were inoculated in 5 mL of SC medium with appropriate selectivity. After transferring, the culture was inoculated in the fermentation medium with an initial OD_600_ = 0.1 for shake flask fermentation. The fermentation medium was YPD medium with diploid sugar (40 g/L of glucose). All strains were grown in shake flasks at 30°C and 150 rpm, and the ratio of flask volume:medium was 5:1. When GAL1p yeast strains completely consumed glucose for growth (24 h), galactose, with a final concentration of 20 g/L, was added to the medium to introduce gene overexpression.

### The Extraction of Vitamin C

Yeast cells of 5 mL fermentation were harvested in a 10-mL ice-cold centrifugal tube by centrifugation at 4°C and 5,000 rpm for 10 min, washed once with ice-cold distilled water in a new ice-cold 2 mL centrifugal tube, and then resuspended in 400 μL ice-cold 2.5% metaphosphate and 100 μL 1 M DTT. The same cell volume of silicon dioxide was added, and the cells were vortexed vigorously for 5 min. Then they were immediately kept in ice for 5 min. After six cycles, the supernatant was then cleared from cell debris by centrifugation at 4°C and 12,000 rpm for 15 min and transferred to a new ice-cold 2-mL centrifugal tube with 15 μL of aqueous ammonia to adjust the pH. The supernatant passed through a 0.22-μm filter prior to high-performance liquid chromatography (HPLC) analyses.

### Vitamin C, Glucose, and Ethanol Quantification

The identity of VC was confirmed by HPLC (Waters e2695–2489) with a Hypersil GOLD^TM^ HILIC column (5 μm inner diameter, 250 μm by 4.6 μm, Thermo Scientific, catalog no. 26505–254630) with 85% acetonitrile, 14.985% H_2_O, and 0.015% phosphoric acid, a flow rate of 1 mL/min, and UV detection set at 245 nm, with pure VC as the standard (as shown in Supplementary Figure S3).

The identity of glucose and ethanol was confirmed by HPLC with an Aminex^®^ HPX-87H Ion Exclusion column (9-μm inner diameter, 300 μm by 7.8 μm, Bio-Rad, catalog no. 125–0140) with 5 mM H_2_SO_4_, a flow rate of 0.6 mL/min, at 65°C with glucose⋅2H_2_O and absolute ethanol as the standard.

### Transcriptional Analysis

RNA was extracted using the Column Fungal RNAout (TIANDZ, catalog no. 80804-50). Nearly 1 μg of total RNA was used for cDNA synthesis with the SPARKscript II RT Kit (SparkJade), following the manufacturer’s instructions. cDNA samples were diluted with ddH_2_O and were used for fluorescence quantitative PCR (qPCR). qPCR analysis was performed on q225–0265 qPCR system (Kubo tech Ltd.) using Unique Aptamer^TM^ qPCR SYBR^®^ Green Master Mix (Novogene). The primers used for qPCR to quantify relevant genes are listed in Supplementary Table S2. The internal standard is Gene TDH3.

## Results

### The Construction and Fermentation of Vitamin C Biosynthesis Strain

**FIGURE 1:**
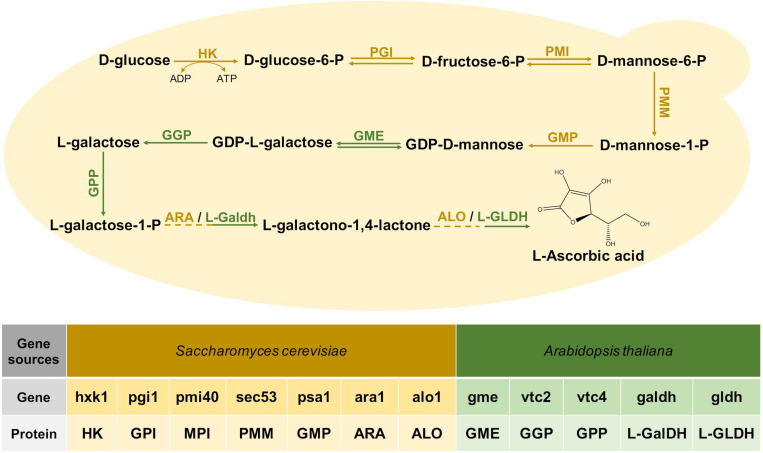
The Vitamin C biosynthesis pathway reconstructed in *S. cerevisiae*. The following enzymes are involved: HK, hexokinase; GPI, glucose-6-phosphate isomerase; MPI, mannose-6-phosphate isomerase; PMM, phosphomannomutase; GMP, GDP-mannose pyrophosphorylase; GME, GDP-mannose-3,5-epimerase; GGP, GDP-L-galactose phosphorylase; GPP, L-Galactose 1-phosphate phosphatase; ARA, D-Arabinose dehydrogenase; ALO, D-Arabinono-1,4-Lactone Oxidase; L-GalDH, L-galactose dehydrogenase; L-GLDH, L-galactono-1,4-lactone dehydrogenase. The introduced genes from *Arabidopsis* are marked in green.

**FIGURE 2:**
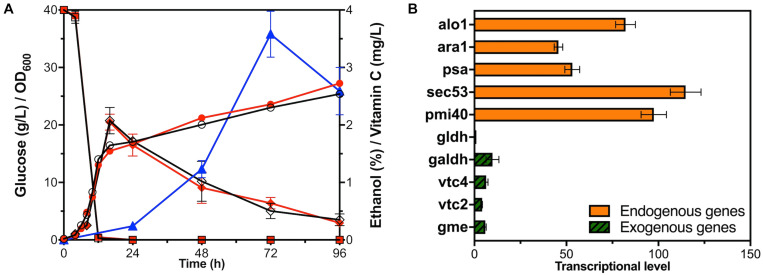
Fermentation and transcriptional level analysis of YLAA. **(A)** Time courses showing changes in cell concentration (circles), glucose consumption (squares), ethanol production (rhombuses), and VC production (triangles) of YLAA (red solid) and BY4741 (black hollow) during fermentation. **(B)** The transcriptional level of genes involved in the VC biosynthetic pathway in strain YLAA. The control gene is Gene gldh. Error bars represent ± standard error of mean (SEM, *n* = 3).

The VC synthesis pathway of plants starts with glucose, and it is finally produced after 10 steps of reactions (Figure 1; Wheeler et al., 1998). Three bio-reactive enzymes of this pathway, GME, GGP, and GPP, did not exist in *S. cerevisiae*, and the research of Branduardi et al. (2007) proved that these genes from *Arabidopsis* could express exogenously. Since codon optimization would improve the efficiency of the enzyme (Wang et al., 2017), we optimized the codons of the genes related to this pathway from *Arabidopsis.* Each gene was collocated with a different constitutive promoter and terminator to construct an exogenous module, and all the modules were introduced to *S. cerevisiae* BY4741, but VC could not be detected. After that, two modules, respectively, expressing L-GalDH, L-GLDH were together transformed into BY4741 resulting in the recombinant stain YLAA. This construction enabled it to synthesize VC from glucose, and it accumulated 3.58 ± 0.40 mg/L of intracellular VC at 72 h (Figure 2A).

In order to investigate whether the introduction of exogenous modules related to VC synthesis would affect the normal cell growth, the curves of the growth, the glucose consumption, and the ethanol and VC production of strain BY4741 and YLAA are indicated in Figure 2A. The growth of YLAA was basically similar to BY4741, and 40 g/L of glucose was simultaneously consumed at the end of the logarithm. Following this, ethanol was consumed for the secondary growth of the strain with the final OD_600_ of about 25 in the stationary phase. The results indicate that the introduction of the VC synthetic pathway did not bring additional growth burden and glucose requirement to *S. cerevisiae*. In addition, intracellular VC began to accumulate at 24 h and reached a maximum at 72 h. The reason for the decrease at 96 h might be due to its oxidation. It could be seen that VC synthesis was not synchronized with the growth in *S. cerevisiae*. It indicated that GDP-D-mannose was first used to synthesize cytoderm to achieve high cell concentration, and then the excess was converted to VC (Jiang et al., 2008).

In order to investigate the rate-limiting steps of the pathway, the transcriptional levels of five endogenous genes and five exogenous genes in YLAA were simultaneously detected (Figure 2B). It could be apparently seen that the transcriptional levels of the five endogenous genes were much higher than those of the exogenous genes. Among the transcriptional levels of endogenous genes, the highest one was sec53, and the lowest two were psa1 and ara1. What was more critical was the comparison of the transcriptional level of exogenous genes. The gene with the highest transcriptional level was galdh, and the lowest two were vtc2 and gldh. The transcriptional level of galdh was about 10-fold that of gldh. The transcriptional level of ara1 was about 46-fold that of gldh. In summary, one of the sticking points to improve the production of VC was how to increase the transcriptional level and expression intensity of exogenous genes, especially vtc2 and gldh.

### Metabolic Pathway Optimization by Engineering Multiple Exogenous Modules

#### The Whole-Exogenous-Module Overexpression

**FIGURE 3:**
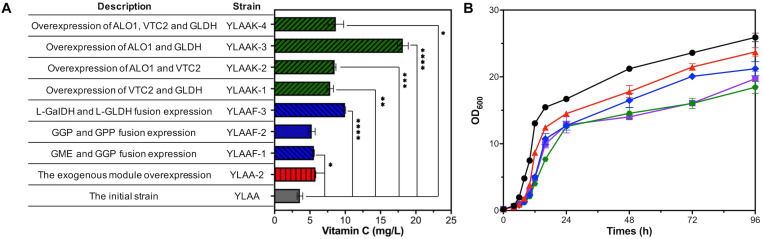
Fermentation of the strains with multiple-exogenous-module optimization. **(A)** The VC production of the initial strain (YLAA, gray), the strain overexpressing the whole exogenous module (YLAA-2, red), the strain overexpressing different fusion-protein (YLAAF-1/2/3, blue), and the strain overexpressing different combination modules (YLAAK-1/2/3/4, green) after 72 h of fermentation. **(B)** Time courses showing changes in cell concentration of YLAAK-1 (triangles), YLAAK-2 (rhombuses), YLAAK-3 (hexagons), YLAAK-4 (squares) and BY4741 (circles). Error bars represent ± SEM (*n* = 3). Significance levels of Student’s *t*-test: ^∗^*P* < 0.05, ^∗∗^*P* < 0.01, ^∗∗∗^*P* < 0.001, and ^****^P < 0.0001.

Improving the transcription levels of exogenous genes was urgently needed, so five exogenous modules involved in the VC pathway were overexpressed by multicopy plasmid to obtain strain YLAA-2. After the shake flask fermentation of YLAA-2, the production of VC was detected at 72 h. Its production was increased by 61.5% compared with the initial strain YLAA, reaching 5.78 ± 0.04 mg/L (Figure 3A).

#### Protein Fusion Engineering

According to the above qPCR results, the rate-limiting enzymes of the VC synthesis pathway in *S. cerevisiae* were probably GPP and L-GLDH. The fusion protein had the complete coding sequence of each enzyme molecule, so it owned the respective catalytic functions of the enzyme molecules that composed it. Therefore, a protein engineering strategy using (GGGGS)_3_ flexible linker for fusion overexpression of GPP or L-GLDH and its adjacent-step protein was adopted, resulting in constructing three recombinant strains (Huston et al., 1988). After fermentation for 72 h, only the fusion overexpression of L-Galdh and L-GLDH significantly increased the intracellular VC accumulation, reaching 9.97 ± 0.09 mg/L, which has been raised 1.78 times as against that of YLAA (Figure 3A). The production increase of the other two strains was not obvious, and it might be caused by the changes in enzyme structure or the inappropriate linker. The main reasons for the increase of strain YLAAF-3 might be the following: Proximity effects were exhibited by the fusion of enzymes in adjacent catalytic reactions, and transient time of reactions was decreased (Ljungcrantz et al., 1989, 1990). In addition, fusion-protein expression could reduce the degradation of intermediate metabolites by the intracellular proteases (Albertsen et al., 2011; Dai et al., 2012; Ignea et al., 2014).

#### Copy Number Engineering of the Possible Rate-Limiting Genes for Its Expression Optimization

It was inferred from qPCR results that it was urgent to increase the expression intensity of vtc2 and gldh. Some studies demonstrated that D-arabinono-1,4-lactone oxidase (ALO) was likely to be an isoenzyme of L-GLDH (Huh et al., 1994; Lee et al., 1999; Sauer et al., 2004). Sauer et al. (2004) found that overexpression of Gene alo1 and gldh enhanced yeast cells to accumulate VC, which indicated the significance of the last step. Three genes are divided into four combination modules: vtc2 and gldh; alo1 and vtc2; alo1 and gldh; and alo1, vtc2, and gldh. Aiming to improve the strength of expression initiation, each gene was equipped with a strong constitutive promoter, e.g., TEF1p, TDH3p, or GPM1p (Partow et al., 2010; Sun et al., 2012; Wang et al., 2015). Thus, four recombinant strains overexpressing the above gene modules were constructed. As shown in Figure 3A, YLAAK-3 had the highest production (16.97 ± 0.65 mg/L), which was consistent with the conclusion that the last step of the VC synthesis pathway was the rate-limiting step (Sauer et al., 2004). The others were similarly about twice that of YLAA.

From the growth curve of strains, the growth of four recombinant strains was obviously weaker than that of BY4741 and YLAA (Figure 3B). Its specific manifestations were long lag phase, lagging logarithmic phase, and low cell concentration. In addition, it was included that the overexpression of alo1 had a significant negative impact on the cell growth. The weakest strains were YLAAK-3 and YLAA-4, and the same genes they overexpressed were alo1 and gldh. In summary, overexpression of excessive gene modules indeed caused the growth and metabolism disorders in *S. cerevisiae*, especially the modules related to the important last step.

### Engineering the Pathway of Vitamin C by Improving Single Exogenous Module Expression

Due to the negative effects of overexpression of excessive gene modules, we attempted to overexpress one exogenous module. In this study, two overexpression methods were chosen: Fusions of an open reading frame to GAL1p inducible promoter; Using plasmid with multiple copies (Rine, 1991).

#### Improving an Exogenous Module Expression by Gal1p

The first step of gene expression is DNA transcription. The promoter is a sequence that RNA polymerase recognizes, binds, and begins to transcribe. Therefore, transcription with a suitable promoter was of considerable significance for metabolic engineering (Nevoigt et al., 2006). The GAL1 promotor initiated induction with galactose as the sole carbon source in the medium and had a strong transcriptional initiation strength (Stagoj and Komel, 2008). According to the above conclusions, VC was beginning to accumulate in *S. cerevisiae* cells when glucose was lacking. Therefore, GAL1p was used to induce the overexpression of target genes by exogenous addition of galactose after glucose has been completely consumed.

**FIGURE 4:**
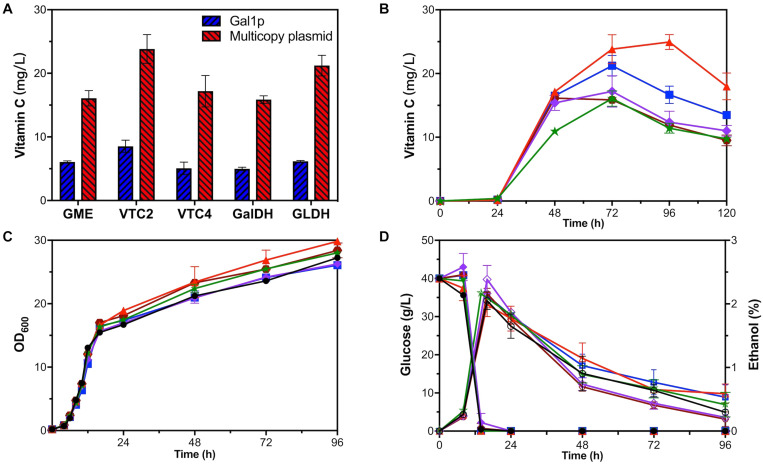
Fermentation of one-gene-module overexpression strains. **(A)** VC production of GAL1p overexpression strains (blue) and multicopy plasmid overexpression strains (red). Time courses showing changes in **(B)** VC production, **(C)** cell concentration, **(D)** glucose consumption (solid), and ethanol production (hollow) of GME-M (green stars), VTC2-M (red triangles), VTC4-M (purple rhombuses), GalDH-M (brown hexagons), GLDH-M (blue squares), and YLAA (black circles).

Each exogenous module, respectively, overexpressing gme, vtc2, vtc4, galdh, and gldh by GAL1p was introduced into strain YLAA to obtain five overexpression strains. Through HPLC results, we found that the use of GAL1p to overexpress any exogenous module indeed increased the intracellular accumulation of VC to varying degrees. Among them, strain VTC2-Gal had the greatest impact on the production (8.53 ± 0.95 mg/L), which indicated that vtc2 is one of the rate-limiting genes in this pathway (Figure 4A). However, the production was not as high as that of the previous strains, and the external addition of galactose caused a supernumerary increase in cost.

#### Balancing Gene Expression and Cell Growth by Copy Number Engineering

In addition to protein engineering, using multicopy plasmid was often used to increase the strength of gene expression in *S. cerevisiae* (Rine, 1991). What is more, increasing the copy numbers of too many gene modules caused disturbances in cell growth and metabolism. Therefore, the copy numbers of only one gene module were increased to improve its cell growth and metabolism.

Five recombinant strains were constructed to investigate the effect of a single gene overexpression by multicopy plasmid on the VC production in *S. cerevisiae*. As shown in Figure 4A, it could be clearly seen that the positive effect of using multicopy for overexpression on the production was better than using GAL1p. The production range was 15.86 ± 0.60–23.83 ± 2.25 mg/L at 72 h. The production of strain VTC2-M was the highest, which has been raised nearly 5.66-fold compared with that in YLAA. As shown in Figure 4B, it could be clearly seen that all strains accumulated VC after 24 h, and the VC accumulation rate was fast from 24 to 48 h. It was worth paying attention that the difference from other strains was that VTC2-M reached the highest value of VC production of 24.94 ± 1.16 mg/L at 96 h (Figure 4B). The results once more indicated the importance of vtc2 in this pathway. From other aspects, overexpression of a single gene module promoted its cell concentration. Interestingly, the ethanol content of the two strains with the highest production was a little higher among five strains (Figures 4C,D). When secondary growth is caused by the consumption of ethanol and other metabolites in the stationary phase, their final OD_600_ was higher than YLAA. In particular, the cell growth of VTC2-M was better than others. The reason could be that higher VC bio-synthesis led to more reduction of reactive oxygen species (ROS) through redox reaction. By comparing the results of Figures 3B, 4C, it was found that their cell concentration was higher than that of strains overexpressing combination modules, which might be due to the reduction of metabolic burden that allowed cells to perform normal physiological activities.

**FIGURE 5:**
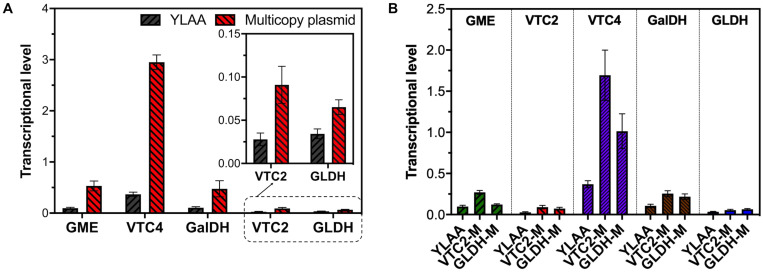
The transcription level of multicopy plasmid overexpression stains. **(A)** The transcription level of corresponding exogenous genes in multicopy plasmid overexpression strains (red) and YLAA (gray). **(B)** The transcription level of exogenous genes, gme (green), vtc2 (red), vtc4 (purple), galdh (brown), and gldh (blue). The first bar of every group is the transcription level of gene in YLAA. The second one is that in VTC2-M. The third one is that in GLDH-M. Error bars represent ± SEM (*n* = 3).

In order to quantify the increment of gene copy number, the transcriptional level of the corresponding gene in YLAA and five overexpression strains was analyzed (Figure 5A). Obviously, the overexpression by multicopy plasmid increased the transcription level by increasing its copy number. The copy number of vtc4 in VTC4-M increased the most, up by eightfold, followed by GME-M and GalDH-M. Both the copy number for gme and galdh almost increased by sixfold. The remaining VTC2-M or GLDH-M only increased by three or twofold, respectively. By combining with the VC production, there was a conclusion: For those genes whose transcriptional level were already relatively high, such as vtc4, gme, and galdh, the increased copy number was not as effective as the overexpression of genes with low transcription level, such as vtc2 and gldh. Furthermore, the strains with a low copy number increase had higher VC accumulation, which might be related to a more appropriate ratio of gene copy numbers. Through the analysis of the transcriptional level of all exogenous genes in VTC2-M, GLDH-M, and YLAA (Figure 5B), it was found that overexpression of vtc2 or gldh not only increased its own transcriptional level but also increased the transcription level of others. It might be related to the accent and decline of the intermediate metabolite level.

### The Effect of Adding Exogenous Substances on Vitamin C Production

In order to investigate which intermediate metabolites were still lacking in the process of the VC synthesis, 250 mg/L of L-galactose or L-galactono-1,4-lactone was exogenously added to the shake flask. What is more, the addition of glutathione (GSH) promoted thiamin/thiamin pyrophosphate (TPP) and GSH transport, increased the metabolism of tricarboxylic acid cycle and pentose phosphate pathway, and upregulated ROS detoxification proteins (Ma et al., 2012). At the same time, it was also an antioxidant, so 200 mg/L of GSH was exogenously added in the hope that it might reduce the loss of VC.

**FIGURE 6:**
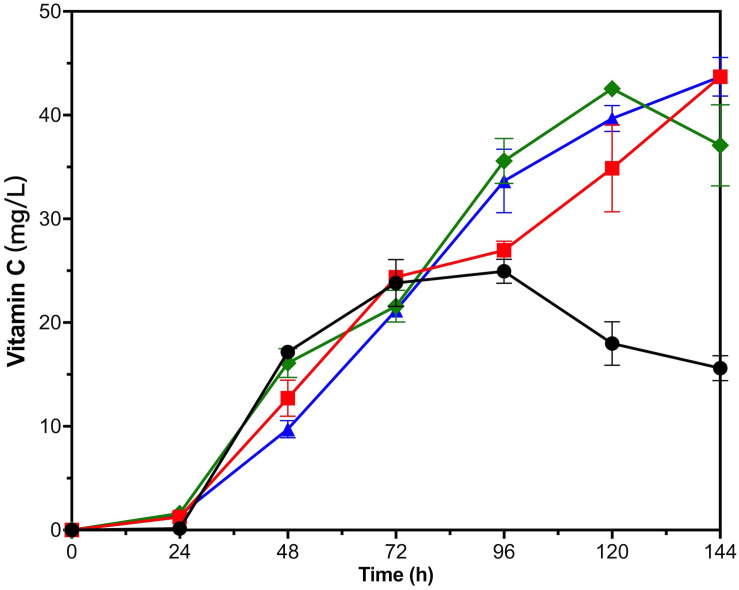
Time courses showing changes in Vitamin C production of VTC2-M with adding exogenous substances. The addition of L-galactose (red squares), L-galactono-1,4-lactone (green rhombuses), GSH (blue triangles), and none (black circles). Error bars represent ± SEM (*n* = 3).

For VTC2-M, all three kinds of exogenous substances had a positive effect on the VC biosynthesis in late fermentation period (Figure 6). VTC2-M with the addition of GSH and L-galactose accumulated nearly 44 mg/L of VC intracellularly on the fifth day, and there seemed to be an upward trend. With the addition of L-galactono-1,4-lactone, the accumulation of VC reached the highest value (42.56 ± 0.65 mg/L) on the fourth day and then declined. Since L-galactose was the upstream intermediate metabolite of L-galactono-1,4-lactone, and the effect of adding this substance was consistent with that of vtc2 overexpression, we inferred that it was critical to reconstruct and even look for more efficient enzymes than GPP encoded by vtc2 from *Arabidopsis*. As for the reason for the positive effect of the addition of GSH, it could be further proved by transcription analysis in the future.

## Discussion

The two-step fermentation method, used in industrial production of VC, included two chemical reaction steps and two biological fermentation steps. Nowadays, people are focusing on achieving one-step fermentation. Branduardi et al. (2007) first designed the metabolic pathway of synthesizing 0.2 mg/L of VC from glucose in *S. cerevisiae*. *S. cerevisiae* cells, as a model organism, were widely used to express exogenous genes for large-scale industrial production due to the well investigation of its genetic and growth characteristics (Lian et al., 2018). However, researchers were currently exploring the stress resistance of VC biosynthesis yeasts (Branduardi et al., 2007; Fossati et al., 2010; Martani et al., 2013). This research focused on exploring the rate-limiting steps and optimizing the VC biosynthesis in *S. cerevisiae*.

Overexpressing alo1 and gldh in several module combinations has the best effect to improve VC production, which emphasized the importance of the last step (Figure 3). Sauer et al. (2004) enhanced the ability of yeast cells to generate VC from L-galactose by overexpressing alo1 and gldh, which proved that the last step was the rate-limiting step. In addition, the results of overexpressing a single gene module proved that that the step catalyzed by GPP encoded by vtc2 was also one of crucial rate-limiting steps (Figures 4A,B).

Protein fusion could implement the balance and optimization of the synthesis pathway by increasing the concentration of enzymes and intermediate metabolites at the specific location. Özaydin et al. (2013) enhanced the biosynthesis of bisabolene through the fusion protein expression. Combining the fusion protein of t3CrGES/Erg20^WW^ and Erg20^WW^ increased the production of geraniol by 2.73 times (Jiang et al., 2017). The intensity of gene expression also played an important role for product synthesis. Kim et al. (2016) regulated gene expression by promoter engineering to achieve low ethanol production and high 2,3-butanediol production. Chen et al. (2012) improved the expression of exogenous proteins by multi-copy plasmids and enhanced patchoulol production. In this study, the metabolic engineering methods were improved and applied to the optimization of one-step biosynthesis of VC in *S. cerevisiae.* First, the fusion expression of L-GalDH and L-GLDH was applied and increased intracellular VC production by 2.78-fold in *S. cerevisiae* (Figure 3A). In order to balance cell production and concentration, a single-module-overexpression strategy was adopted. The best recombinant strain overexpressing vtc2 could accumulate 24.94 ± 1.16 mg/L of intracellular VC at 96 h, and it could reach nearly 44 mg/L in 144 h with the exogenous addition of 250 mg/L of L-galactose or 200 mg/L GSH (Figures 4, 6). Our research provided the experience in engineering *S. cerevisiae* strains for one-step fermentation of VC or any other valuable chemical production.

One-step biosynthesis of VC by *S. cerevisiae* has been long sought for, but there is still a long way to go. What is more, a yeast capable of synthesizing VC improved its antioxidant and the tolerance to low pH, weak organic acids, and the positive effect of VC on the treatment of cancer was related to its influence on ROS (Branduardi et al., 2007; Yun et al., 2015). The recombinant yeast constructed in this study could also represent a cellular model to investigate the occurrence/protection of ROS in eukaryote.

## Data Availability Statement

The original contributions presented in the study are included in the article/Supplementary Material, further inquiries can be directed to the corresponding author/s.

## Author Contributions

MZ and YB performed the experiment together, analyzed the data, and wrote the first draft. MD is corresponding joint authors, was responsible for the manuscript writing, designed and guided this study, provided project funding, and revised the manuscript. YY participated in the design of the study and put forward constructive suggestions for solving problems. All authors contributed to the article and approved the final manuscript.

## Conflict of Interest

The authors declare that the research was conducted in the absence of any commercial or financial relationships that could be construed as a potential conflict of interest.
