# Supplementary material for: One-Step Biosynthesis of Vitamin C in Saccharomyces cerevisiae
Source: Front Microbiol. 2021 Feb 25;12:643472. doi: 10.3389/fmicb.2021.643472 (PMC7947327; doi:10.3389/fmicb.2021.643472)
Supplement: Supplementary file 1 [file Data_Sheet_1.PDF]

Table S1. Primers used in this study.

Plasmid	Fragments	Primers	Sequence (5'-3')
p2-4-6-1	PGK1p	PGK1p-F1	CACTATAGGGCGAATTGGGTACCGGGCCCCCCCTCGAGGTCGACGGTATCGATAAGCTTTATTTTAGATTCCTGACTTCAACTCAAGAC
		PGK1p-R1	TACCATTTGTAGTACCCATTGTTTTATATTTGTTGTAAAAAGTAGATAATTACTTCCTT
	gme	gme-F2	TTTACAACAAATATAAAACAATGGGTACTACAAATGGTACTGATTAC
		gme-R2	TTAATAAAAGTGTTCGCAAATTATTCTTTACCATCAGCAGCTCTTAATG
	HXT7t	HXT7t-F3	CTGCTGATGGTAAAGAATAATTTGCGAACACTTTTATTAATTCATGATCAC
		HXT7t-R3	CTTCCTGCTCACAAATCTTAAAGTCATACATTGCACGACTAGAGACCGGTCTCGGACTAATAACTGACTCATTAGACACTTTTTGAAGC
	GPM1p	GPM1p-F4	TTCTGTATCCCGCTTCAAAAAGTGTCTAATGAGTCAGTTATTAGTCCGAGACCGGTCTCTAGTCGTGCAATGTATGACTTTAAGATTTG
		GPM1p-R4	ACTCTTTTAATCTTCAACATTATTGTAATATGTGTGTTTGTTTGGATTATTAAGAA
	vtc2	vtc2-F5	CAAACACACATATTACAATAATGTTGAAGATTAAAAGAGTTCCAACAGTTG
		vtc2-R5	GTGACATAACTAATTACATGTTATTGCAAAACTAAACATTCAGCAGCAG
	CYC1t	CYC1t -F6	AATGTTTAGTTTTGCAATAACATGTAATTAGTTATGTCACGCTTACATTC
		CYC1t-R6	TTAATTATTTACGTATTCTTTGAAATGGCAGTATTGATAATGATAAACTGAGACCGGTCTCGAAACTAAAGCCTTCGAGCGTCCCAAAA
	TDH3p	TDH3p-F7	AACCTTGCTTGAGAAGGTTTTGGGACGCTCGAAGGCTTTAGTTTCGAGACCGGTCTCAGTTTATCATTATCAATACTGCCATTTCAAAG
		TDH3p-R7	AATTGATCATTATCTGCCATTTTGTTTGTTTATGTGTGTTTATTCGAAACTAAG
	vtc4	vtc4-F8	AACACACATAAACAAACAAAATGGCAGATAATGATCAATTTTTGGCTG
		vtc4-R8	AAATCATTCATTCTTCAGACTTAAGCACCTGTTAATCTCAATGCTTCT
	GPM1t	GPM1t-F9	TGAGATTAACAGGTGCTTAAGTCTGAAGAATGAATGATTTGATGATTTCT
		GPM1t-R9	GAAGCGCCTACGCTTGACATCTACTATATGTAAGTATACGGCCCCGAGACCGGTCTCGGCCCCTATTCGAACTGCCCATTCAGCTTTTC
	TEF2p	TEF2p-F10	GAGTGCACCAATTGCAAAGGGAAAAGCTGAATGGGCAGTTCGAATAGGGGCCGAGACCGGTCTCGGGGCCGTATACTTACATATAGTAG
		TEF2p-R10	AACAACAAAGATCTCAACATGTTTAGTTAATTATAGTTCGTTGACCGTATATTCTAAAAAC
	gldh	gldh-F11	CGAACTATAATTAACTAAACATGTTGAGATCTTTGTTGTTGAGGAGG
		gldh-R11	AATTCTTAGTTAAAAGCACTTTAAGCAGTAGTAGAGACTGGGAACA
	ENO2t	ENO2t-F12	CAGTCTCTACTACTGCTTAAAGTGCTTTTAACTAAGAATTATTAGTCTTTTCTGC
		ENO2t-R12	ATAGAGGAAGCTGAAAAGTCTTAGAACGGGTAATCTTCCACCAACCTGATGGGTTCCTAGATATAAGGTATCATCTCCATCTCCCATATG
	TPI1p	TPI1p-F13	GATGGAAGGAAATGCGGGCCACGACCACAGTGATATGCATATGGGAGATGGAGATGATACCTTATATCTAGGAACCCATCAGGTTGGTG
		TPI1p-R13	TTAATTCAATCTTTGTCATTTTTAGTTTATGTATGTGTTTTTTGTAGTTATAGATTTAAGCAA
	galdh	galdh-F14	AACACATACATAAACTAAAAATGACAAAGATTGAATTAAGGGCTTTGG
		galdh-R14	CGATTTCAATTCAATTCAATTTAGTTCTGGTGAATACCAGATGGC
	PGK1t	PGK1t-F15	CTGGTATTCACCAGAACTAAATTGAATTGAATTGAAATCGATAGATCAATTTTTTTCTT
		PGK1t-R15	AGCTCCACCGCGGTGGCGGCCGCTCTAGAACTAGTGGATCCCCCGGGCTGCAGGAATTCAACGAACGCAGAATTTTCGAGTTATTAAAC

Table S1（continue）

Plasmid	Fragments	Primers	Sequence (5'-3')
	PGK1p-gme-HXT7t	PGK1p-F1	CACTATAGGGCGAATTGGGTACCGGGCCCCCCCTCGAGGTCGACGGTATCGATAAGCTTTATTTTAGATTCCTGACTTCAACTCAAGAC
		HXT7t-R3	CTTCCTGCTCACAAATCTTAAAGTCATACATTGCACGACTAGAGACCGGTCTCGGACTAATAACTGACTCATTAGACACTTTTTGAAGC
	GPM1p-vtc2-CYC1t	GPM1p-F4	TTCTGTATCCCGCTTCAAAAAGTGTCTAATGAGTCAGTTATTAGTCCGAGACCGGTCTCTAGTCGTGCAATGTATGACTTTAAGATTTG
		CYC1t-R6	TTAATTATTTACGTATTCTTTGAAATGGCAGTATTGATAATGATAAACTGAGACCGGTCTCGAAACTAAAGCCTTCGAGCGTCCCAAAA
	TDH3p-vtc4-GMP1t	TDH3p-F7	AACCTTGCTTGAGAAGGTTTTGGGACGCTCGAAGGCTTTAGTTTCGAGACCGGTCTCAGTTTATCATTATCAATACTGCCATTTCAAAG
		GPM1t-R9	GAAGCGCCTACGCTTGACATCTACTATATGTAAGTATACGGCCCCGAGACCGGTCTCGGCCCCTATTCGAACTGCCCATTCAGCTTTTC
	TEF2p-gldh-ENO2t	TEF2p-F10	GAGTGCACCAATTGCAAAGGGAAAAGCTGAATGGGCAGTTCGAATAGGGGCCGAGACCGGTCTCGGGGCCGTATACTTACATATAGTAG
		ENO2t-R12	ATAGAGGAAGCTGAAAAGTCTTAGAACGGGTAATCTTCCACCAACCTGATGGGTTCCTAGATATAAGGTATCATCTCCATCTCCCATATG
	TPI1p-galdh-PGK1t	TPI1p-F13	GATGGAAGGAAATGCGGGCCACGACCACAGTGATATGCATATGGGAGATGGAGATGATACCTTATATCTAGGAACCCATCAGGTTGGTG
		PGK1t-R15	AGCTCCACCGCGGTGGCGGCCGCTCTAGAACTAGTGGATCCCCCGGGCTGCAGGAATTCAACGAACGCAGAATTTTCGAGTTATTAAAC
pGLDH-1/2	TEF2p-gldh-ENO2t	NotI-TEF2p-F	AAGGAAAAAAGCGGCCGCGGGGCCGTATACTTACATATAGTAG
		ENO2t-SacI-R	CGAGCTCAGGTATCATCTCCATCTCCCATATG
p2-4-6-1-2	L-PGK1p-gme-HXT7t	L-F	AGGGTTGAGTGTTGTTCCAGTT
		HXT7t-R3	CTTCCTGCTCACAAATCTTAAAGTCATACATTGCACGACTAGAGACCGGTTCGGACTAATAACTGACTCATTAGACACTTTTTGAAGC
	HXT7t-GPM1p-vtc2-CYC1t	HXT7t-F3	CTGCTGATGGTAAAGAATAATTTGCGAACACTTTTATTAATTCATGATCAC
		CYC1t-R6	TTAATTATTTACGTATTCTTTGAAATGGCAGTATTGATAATGATAAACTGAGACCGGTCTCGAAACTAAAGCCTTCGAGCGTCCCAAAA
	CYC1t-TDH3p-vtc4-GPM1t	CYC1t-F6	AATGTTTAGTTTTGCAATAACATGTAATT AGTTATGTCACGCTTACATTC
		GPM1t-R9	GAAGCGCCTACGCTTGACATCTACTATATGTAAGTATACGGCCCCGAGACCGGTCTCGGCCCCTATTCGAACTGCCCATTCAGCTTTTC
	GPM1t	GPM1t-F9	TGAGATTAACAGGTGCTTAAGTCTGAAGAATGAATGATTTGATGATTTCT
		GPM1t-R9	GAAGCGCCTACGCTTGACATCTACTATATGTAAGTATACGGCCCCGAGACCGGTCTCGGCCCCTATTCGAACTGCCCATTCAGCTTTTC
	TEF2p	TEF2p-F10	GAGTGCACCAATTGCAAAGGGAAAAGCTGAATGGGCAGTTCGAATAGGGGCCGAGACCGGTCTCGGGGCCGTATACTTACATATAGTAG
		TEF2p-R10	AACAACAAAGATCTCAACATGTTTAGTTAATTATAGTTCGTTGACCGTATATTCTAAAAAC
	GPM1t-TEF2p	GPM1t-F9	TGAGATTAACAGGTGCTTAAGTCTGAAG AATGAATGATTTGATGATTTCT
		TEF2p-R10	AACAACAAAGATCTCAACATGTTTAGTTAATTATAGTTCGTTGACCGTATATTCTAAAAAC

Table S1（continue）

Plasmid	Fragments	Primers	Sequence (5'-3')
	TEF2p-gldh-ENO2t	TEF2p-R10	AACAACAAAGATCTCAACATGTTTAGTTAATTATAGTTCGTTGACCGTATATTCTAAAAAC
		ENO2t-R12	ATAGAGGAAGCTGAAAAGTCTTAGAACGGGTAATCTTCCACCAACCTGATGGGTTCCT AGATATAAGGTATCATCTCCATCTCCCATATG
	ENO2t-TPI1p-galdh-PGK1t-R	ENO2t-F12	CAGTCTCTACTACTGCTTAAAGTGCTTTTAACTAAGAATTATTAGTCTTTTCTGC
		R-R	CTGCGTTATCCCCTGATTCTGT
pGME-VTC2	L-GPM1p-vtc2	L-F	AGGGTTGAGTGTTGTTCCAGTT
		vtc2-gme-R	AGAACCACCACCACCAGAACCACCACCACCAGAACCACCACCACCTTGCAAAACTAA ACATTCAGCAGCAG
	vtc2(gme)	vtc2-F5	CAAACACACATATTACAATAATGTTGAAGAT TAAAAGAGTTCCAACAGTTG
		vtc2-gme-R	AGAACCACCACCACCAGAACCACCACCACCAGAACCACCACCACCTTGCAAAACTAA ACA TTCAGCAGCAG
	(vtc2)gme	vtc2-gme-F	GGTGGTGGTGGTTCTGGTGGTGGTGGTTCTGGTGGTGGTGGTTCTGGTACTACAAAT GGTACTGATTACGGT
		gme-R2	TTAATAAAAGTGTTCGCAAATTATTCTTTACC ATCAGCAGCTCTTAATG
	vtc2-gme	vtc2-F5	CAAACACACATATTACAATAATGTTGAAGAT TAAAAGAGTTCCAACAGTTG
		gme-R2	TTAATAAAAGTGTTCGCAAATTATTCTTTACC ATCAGCAGCTCTTAATG
	gme(CYC1t)	vtc2-gme-F	GGTGGTGGTGGTTCTGGTGGTGGTGGTTCTGGTGGTGGTGGTTCTGGTACTACAAAT GGTACTGATTACGGT
		gme(CYC1t)-R	GTGACATAACTAATTACATGTTATTCTTTACC ATCAGCAGCTCTTAATGAAC
	(gme)CYC1t	gme(CYC1t)-F	CTGCTGATGGTAAAGAATAACATGTAATTAG TTATGTCACGCTTACATTC
		CYC1t-R6	TTAATTATTTACGTATTCTTTGAAATGGCAGTATTGATAATGATAAACTGAGACCGGTCTC GA AACTAAAGCCTTCGAGCGTCCCAAAA
	gme-CYC1t	vtc2-gme-F	GGTGGTGGTGGTTCTGGTGGTGGTGGTTCTGGTGGTGGTGGTTCTGGTACTACAAAT GGTACTGATTACGGT
		CYC1t-R6	TTAATTATTTACGTATTCTTTGAAATGGCAGTATTGATAATGATAAACTGAGACCGGTCTC GA AACTAAAGCCTTCGAGCGTCCCAAAA
	L-GPM1p-vtc2-gme-CYC1t	L-F	AGGGTTGAGTGTTGTTCCAGTT
		CYC1t-R6	TTAATTATTTACGTATTCTTTGAAATGGCAGTATTGATAATGATAAACTGAGACCGGTCTC GA AACTAAAGCCTTCGAGCGTCCCAAAA
	CYC1t-TDH3p-vtc4-GPM1t	CYC1t-F6	AATGTTTAGTTTTGCAATAACATGTAATTAGTTATGTCACGCTTACATTC
		GPM1t-R9	GAAGCGCCTACGCTTGACATCTACTATATGTAAGTATACGGCCCCGAGACCGGTCTC GGCCCCTATTCGAACTGCCCATTCAGCTTTTC
	GPM1t-TEF2p	GPM1t-F9	TGAGATTAACAGGTGCTTAAGTCTGAAG AATGAATGATTTGATGATTTCT
		TEF2p-R10	AACAACAAAGATCTCAACATGTTTAGTTAATTATAGTTCGTTGACCGTATATTCTAAAA AC
	TEF2p-gldh-ENO2t	TEF2p-R10	AACAACAAAGATCTCAACATGTTTAGTTAATTATAGTTCGTTGACCGTATATTCTAAAAAC
		ENO2t-R12	ATAGAGGAAGCTGAAAAGTCTTAGAACGGGTAATCTTCCACCAACCTGATGGGTTCCT AGATATAAGGTATCATCTCCATCTCCCATATG

Table S1（continue）

Plasmid	Fragments	Primers	Sequence (5'-3')
	ENO2t-TPI1p-galdh-PGK1t-R	ENO2t-F12	CAGTCTCTACTACTGCTTAAAGTGCTTTTAACTAAGAATTATTAGTCTTTTCTGC
		R-R	CTGCGTTATCCCCTGATTCTGT
pVTC2-VTC4	L-PGK1p-gme-HXT7t	L-F	AGGGTTGAGTGTTGTTCCAGTT
		HXT7t-R3	CTTCCTGCTCACAAATCTTAAAGTCATACATTGCACGACTAGAGACCGGTTCGGACTAATAACTGACTCATTAGACACTTTTTGAAGC
	HXT7t-GPM1p-vtc2	HXT7t-F3	CTGCTGATGGTAAAGAATAATTTGCGAACACTTTTATTAATTCATGATCAC
		vtc2-vtc4-R	AGAACCACCACCACCAGAACCACCACCACCAGAACCACCACCACCTTGCAAAACTAAACATTCAGCAGCAGT
	vtc2(vtc4)	vtc2-F5	CAAACACACATATTACAATAATGTTGAAGATTAAAAGAGTTCCAACAGTTG
		vtc2-vtc4-R	AGAACCACCACCACCAGAACCACCACCACCAGAACCACCACCACCTTGCAAAACTAAACATTCAGCAGCAGT
	(vtc2)vtc4	vtc2-vtc4-F	GGTGGTGGTGGTTCTGGTGGTGGTGGTTCTGGTGGTGGTGGTTCTGCAGATAATGATCAATTTTTGGCTGC
		vtc4-R8	AAATCATTCATTCTTCAGACTTAAGCACCTGTTAATCTCAATGCTTCT
	vtc2-vtc4	vtc2-F5	CAAACACACATATTACAATAATGTTGAAGATTAAAAGAGTTCCAACAGTTG
		vtc4-R8	AAATCATTCATTCTTCAGACTTAAGCACCTGTTAATCTCAATGCTTCT
	vtc4-GPM1t	vtc2-vtc4-F	GTGGTGGTGGTTCTGCAGATAATGATCAATT TTTGGCTGC
		GPM1t-R9	GAAGCGCCTACGCTTGACATCTACTATATGTAAGTATACGGCCCCGAGACCGGTCTCGGCCCCTATTCGAACTGCCCATTCAGCTTTTC
	GPM1t-TEF2p	GPM1t-F9	TGAGATTAACAGGTGCTTAAGTCTGAAG AATGAATGATTTGATGATTTCT
		TEF2p-R10	AACAACAAAGATCTCAACATGTTTAGTTAATTATAGTTCGTTGACCGTATATTCTAAAAAC
	TEF2p-gldh-ENO2t	TEF2p-R10	AACAACAAAGATCTCAACATGTTTAGTTAATTATAGTTCGTTGACCGTATATTCTAAAAAC
		ENO2t-R12	ATAGAGGAAGCTGAAAAGTCTTAGAACGGGTAATCTTCCACCAACCTGATGGGTTCCTAGATATAAGGTATCATCTCCATCTCCCATATG
	ENO2t-TPI1p-galdh-PGK1t-R	ENO2t-F12	CAGTCTCTACTACTGCTTAAAGTGCTTTTAACTAAGAATTATTAGTCTTTTCTGC
		R-R	CTGCGTTATCCCCTGATTCTGT
pGalDH-GLDH	L-PGK1p-gme-HXT7t	L-F	AGGGTTGAGTGTTGTTCCAGTT
		HXT7t-R3	CTTCCTGCTCACAAATCTTAAAGTCATACATTGCACGACTAGAGACCGGTTCGGACTAATAACTGACTCATTAGACACTTTTTGAAGC
	HXT7t-GPM1p-vtc2-CYC1t	HXT7t-F3	CTGCTGATGGTAAAGAATAATTTGCGAACACTTTTATTAATTCATGATCAC
		CYC1t-R6	TTAATTATTTACGTATTCTTTGAAATGGCAGTATTGATAATGATAAACTGAGACCGGTCTCGAAACTAAAGCCTTCGAGCGTCCCAAAA
	CYC1t-TDH3p-vtc4-GPM1t	CYC1t-F6	AATGTTTAGTTTTGCAATAACATGTAATTAGTTATGTCACGCTTACATTC
		GPM1t-R9	GAAGCGCCTACGCTTGACATCTACTATATGTAAGTATACGGCCCCGAGACCGGTCTCGGCCCCTATTCGAACTGCCCATTCAGCTTTTC
	GPM1t-TPI1p-galdh	GPM1t-F9	TGAGATTAACAGGTGCTTAAGTCTGAAGAAT GAATGATTTGATGATTTCT
		galdh-gldh-R	AGAACCACCACCACCAGAACCACCACCACCAGAACCACCACCACCAGCAGTAGTAGAGACTGGGAACAAT

Table S1（continue）

Plasmid	Fragments	Primers	Sequence (5'-3')
	galdh(gldh)	galdh-F14	AACACATACATAAACTAAAAATGACAAAGATTGAATTAAGGGCTTTGG
		galdh-gldh-R	AGAACCACCACCACCAGAACCACCACCACCAGAACCACCACCACCAGCAGTAGTAGAGACTGGGAACAAT
	(galdh)gldh	galdh-gldh-F	GGTGGTGGTGGTTCTGGTGGTGGTGGTTCTGGTGGTGGTGGTTCTACAAAGATTGAATTAAGGGCTTTGGG
		gldh-R11	AATTCTTAGTTAAAAGCACTTTAAGCAGTAGTAGAGACTGGGAACA
	galdh-gldh	galdh-F14	AACACATACATAAACTAAAAATGACAAAGATTGAATTAAGGGCTTTGG
		gldh-R11	AATTCTTAGTTAAAAGCACTTTAAGCAGTAGTAGAGACTGGGAACA
	gldh-ENO2t	TEF2p-R10	AACAACAAAGATCTCAACATGTTTAGTTAATTATAGTTCGTTGACCGTATATTCTAAAAAC
		ENO2t-R12	ATAGAGGAAGCTGAAAAGTCTTAGAACGGGTAATCTTCCACCAACCTGATGGGTTCCTAGATATAAGGTATCATCTCCATCTCCCATATG
	gldh-ENO2t-R	galdh-gldh-F	GGTGGTGGTGGTTCTGGTGGTGGTGGTTCTGGTGGTGGTGGTTCTACAAAGATTGAATTAAGGGCTTTGGG
		R-R	CTGCGTTATCCCCTGATTCTGT
pVTC2-GLDH	TEF1p	TEF1p-F	AATTGGGTACCGGGCCCCCCCTCGAGCGCTCGAAGGCTTTAGATCTAATGT
		TEF1p-R	ACTCTTTTAATCTTCAACATTTTGTAATTAAAACTTAGATTAGATTGCTATGCTTTCT
	vtc2-CYC1t	TEF1p-vtc2-F	ATCTAAGTTTTAATTACAAAATGTTGAAGATTAAAAGAGTTCCAACAGTTG
		CYC1t-gldh-R	GATAATGATAAACTGAGACCCTAAAGCCTTCGAGCGTCCC
	TDH3p	TDH3p-F8	GGGACGCTCGAAGGCTTTAGGGTCTCAGTTTATCATTATCAATACTGCCAT
		TDH3p-R8	AACAACAAAGATCTCAACATTTTGTTTGTTTATGTGTGTTTATTCGAAACTAAGTTC
	gldh-ENO2t	CYC1t-gldh-F	AACACACATAAACAAACAAAATGTTGAGATCTTTGTTGTTGAGGAGG
		CYC1t-gldh-R	ATATCAAGCTTATCGATACCGTCGACAGGTATCATCTCCATCTCCCATATGC
	TEF1p-vtc2-CYC1t	TEF1p-F	AATTGGGTACCGGGCCCCCCCTCGAGCGCTCGAAGGCTTTAGATCTAATGT
		CYC1t-gldh-R	GATAATGATAAACTGAGACCCTAAAGCCTTCGAGCGTCCC
	TDH3p-gldh-ENO2t	TDH3p-F8	GGGACGCTCGAAGGCTTTAGGGTCTCAGTTTATCATTATCAATACTGCCAT
		CYC1t-gldh-R	ATATCAAGCTTATCGATACCGTCGACAGGTATCATCTCCATCTCCCATATGC
	vtc2-gldh	TEF1p-F	AATTGGGTACCGGGCCCCCCCTCGAGCGCTCGAAGGCTTTAGATCTAATGT
		ENO2t-R13	ATATCAAGCTTATCGATACCGTCGACAGGTATCATCTCCATCTCCCATATGC
pALO1-VTC2	GPM1p	GPM1p-F5	AATTGGGTACCGGGCCCCCCCTCGAGTAGTCGTGCAATGTATGACTTTAAGATTTGTG
		GPM1p-R5	TGGGATAGTAGACATTATTGTAATATGTGTGTTTGTTTGGATTATTAAGAAGAATAAT
	alo1	alo1-F	CCAAACAAACACACATATTACAATAATGTCTACTATCCCATTTAGAAAGAACTATGTGT
		alo1-R	TTAATAAAAGTGTTCGCAAACTAGTCGGACAACTCACTAGGATCTATAATACC
	HXT7t	alo1-HXT7t-F	CTAGTGAGTTGTCCGACTAGTTTGCGAACACTTTTATTAATTCATGATCACG
		alo1-HXT7t-R	AGATCTAAAGCCTTCGAGCGCTAATAACTGACTCATTAGACACTTTTTGAAGCG
	vtc2	HXT7p-vtc2-F	TCTAATGAGTCAGTTATTAGCGCTCGAAGGCTTTAGATCTAATGT
		CYC1t-425-R	GGCTGCAGGAATTCGATATCAAGCTTCTAAAGCCTTCGAGCGTCCC
	GPM1p-ALO1	GPM1p-F5	AATTGGGTACCGGGCCCCCCCTCGAGTAGTCGTGCAATGTATGACTTTAAGATTTGTG
		ALO1-R	TTAATAAAAGTGTTCGCAAACTAGTCGGACAACTCACTAGGATCTATAATACC
	HXT7p-vtc2	HXT7p-F	CTAGTGAGTTGTCCGACTAGTTTGCGAACACTTTTATTAATTCATGATCACG
		CYC1t-425-R	GGCTGCAGGAATTCGATATCAAGCTTCTAAAGCCTTCGAGCGTCCC

Table S1（continue）

Plasmid	Fragments	Primers	Sequence (5'-3')
	alo1-vtc2	GPM1p-F5	AATTGGGTACCGGGCCCCCCCTCGAGTAGTCGTGCAATGTATGACTTTAAGATTTGTG
		CYC1t-425-R	GGCTGCAGGAATTCGATATCAAGCTTCTAAAGCCTTCGAGCGTCCC
pALO1-GLDH	alo1	GPM1p-F6	AATTGGGTACCGGGCCCCCCCTCGAGTAGTCGTGCAATGTATGACTTTAAGATTTGTG
		HXT7t-TDH3p-R	GATAATGATAAACTGAGACCCTAATAACTGACTCATTAGACACTTTTTGAAGCG
	gldh	HXT7t-TDH3p-F	TCTAATGAGTCAGTTATTAGGGTCTCAGTTTATCATTATCAATACTGCCAT
		ENO2t-425-R	GGCTGCAGGAATTCGATATCAAGCTTAGGTATCATCTCCATCTCCCATATGC
	alo1-gldh	GPM1p-F6	AATTGGGTACCGGGCCCCCCCTCGAGTAGTCGTGCAATGTATGACTTTAAGATTTGTG
		ENO2t-425-R	GGCTGCAGGAATTCGATATCAAGCTTAGGTATCATCTCCATCTCCCATATGC
pALO1	TDH3p	TDH3p-F9	AATTGGGTACCGGGCCCCCCCTCGAGGGTCTCAGTTTATCATTATCAATACTGCCAT
		TDH3p-R9	CTAAATGGGATAGTAGACATTTTGTTTGTTTATGTGTGTTTATTCGAAACT
	alo1	TDH3p-alo1-F	AACACACATAAACAAACAAAATGTCTACTATCCCATTTAGAAAGAACTATGTGT
		TDH3p-alo1-R	AATTCTTAGTTAAAAGCACTCTAGTCGGACAACTCACTAGGATCTATAATAC
	ENO2t	alo1-ENO2t-F	CTAGTGAGTTGTCCGACTAGAGTGCTTTTAACTAAGAATTATTAGTCTTTTCTGCT
		alo1-ENO2t-R	ATATCAAGCTTATCGATACCGTCGACAGGTATCATCTCCATCTCCCATATGC
	TDH3p-alo1-ENO2t	TDH3p-F9	AATTGGGTACCGGGCCCCCCCTCGAGGGTCTCAGTTTATCATTATCAATACTGCCAT
		alo1-ENO2t-R	ATATCAAGCTTATCGATACCGTCGACAGGTATCATCTCCATCTCCCATATGC
pHE-GAL	GAL1p	GAL1p-F1	CCGGAATTCTGGAACTTTCAGTAATACGCTTAACTGC
		GAL1p-R1	CGATTTGTGAGACCGGTCTCTATAGTTTTTTCTCCTTGACGTTAAAGTATAGAGGTATATTAAC
	PGI1t	PGI1t-F2	AAACTATAGAGACCGGTCTCACAAATCGCTCTTAAATATATACCTAAAGAACATTAAAGC
		PGI1t -R2	CGCGGATCCGTAGTTTAGTGTTTTTCTTCCAGTGCG
	GAL1p-PGI1t	GAL1p-F1	CCGGAATTCTGGAACTTTCAGTAATACGCTTAACTGC
		PGI1t -R2	CGCGGATCCGTAGTTTAGTGTTTTTCTTCCAGTGCG
pGME-GAL	(GAL1p) gme (PGI1t)	GAL-gme-F	GGTCTCCCTATAATGGGTACTACAAATGGTACTGATTACGG
		GAL-gme-R	GGTCTCGTTTGTTTATTCTTTACCATCAGCAGCTCTTAATGAAC
pVTC2-GAL	(GAL1p) vtc2 (PGI1t)	GAL-vtc2-F	GGTCTCCCTATAATGTTGAAGATTAAAAGAGTTCCAACAGTTGT
		GAL-vtc2-R	GGTCTCGTTTGTTTATTGCAAAACTAAACATTCAGCAGCAG
pVTC4-GAL	(GAL1p) vtc4 (PGI1t)	GAL-vtc4-F	GGTCTCCCTATAATGGCAGATAATGATCAATTTTTGGCTG
		GAL-vtc4-R	GGTCTCGTTTGTTTAAGCACCTGTTAATCTCAATGCTTCT
pGalDH-GAL	(GAL1p) galdh (PGI1t)	GAL-galdh-F	GGTCTCCCTATAATGACAAAGATTGAATTAAGGGCTTTGGG
		GAL-galdh-R	GGTCTCGTTTGTTTAGTTCTGGTGAATACCAGATGGCC
pGLDH-GAL	(GAL1p) gldh (PGI1t)	GAL-gldh-F	GGTCTCCCTATAATGTTGAGATCTTTGTTGTTGAGGAGG
		GAL-gldh-R	GGTCTCGTTTGTTTAAGCAGTAGTAGAGACTGGGAACAAT
pGME-425	PGK1p-gme-HXT7t	2-gme-F	AAGGAAAAAAGCGGCCGCTATTTTAGATTCCTGACTTCAACTCAAGAC
		2-gme-R	CATAACTGACTCATTAGACACTTTTTGAAGC
pVTC2-425	GPM1p-vtc2-HXT7t	2-vtc2-F	AAGGAAAAAAGCGGCCGCTAGTCGTGCAATGTATGACTTTAAGATTTG
		2-vtc2-R	CAAAGCCTTCGAGCGTCCCAAAA
pVTC4-425	TDH3p-vtc4-GPM1t	2-vtc4-F	AAGGAAAAAAGCGGCCGCAGTTTATCATTATCAATACTGCCATTTCAAAG
		2-vtc4-R	CTATTCGAACTGCCCATTCAGCTTTTC

Table S1（continue）

Plasmid	Fragments	Primers	Sequence (5’-3’)
pGalDH-425	TPI1p-galdh-PGK1t	2-galdh-F	AAGGAAAAAAGCGGCCGCTATATCTAGGAACCCATCAGGTTGGT
		2-galdh-R	CAACGAACGCAGAATTTTCGAGTTATTAAACTTAA
pGLDH-425	TEF2p-gldh-ENO2t	2-gldh-F	AAGGAAAAAAGCGGCCGCGGGGCCGTATACTTACATATAGTAGATGTCAAGCGTAGGCGCTTC
		2-gldh-R	CAGGTATCATCTCCATCTCCCATATGC

Table S2. Primers used for qPCR.

Gene	Primers	Sequence (5'-3')
PMI40	pmi40-qPCR-F	CGCTGTGACTGACTTTGAAG
	pmi40-qPCR-R	TGTTTTGCTCATCTTCTGGGG
SEC50	sce53-qPCR-F	GGGGAAAACGTCGAAAGAGAT
	sce53-qPCR-R	TGGTATGATCAACGTTTCCCC
PSA1	psa1-qPCR-F	GGAGGAAATCTTAGCGGTTGG
	psa1-qPCR-R	GGTTTCTGGATGGATGTTGGT
ARA1	ara1-qPCR-F	AGCCTCAACCGAAAACATAGTC
	ara1-qPCR-R	GGCCCAAGCAGTATCAATGT
ALO1	alo-qPCR-F	GGATAGAGTAACCTTTCGCACC
	alo-qPCR-R	CGGGCCATTCTCCTAGTAAC
GME	gme-qPCR-F	GAAAAATTGGGTTGGGCTCC
	gme-qPCR-R	CAGCAGCTCTTAATGAACCC
VTC2	vtc2-qPCR-F	GGTGCTTGTTGTTTGAATGGTG
	vtc2-qPCR-R	CTATCTTCCCATTCACCCAAAACT
VTC4	vtc4-qPCR-F	TTGGCTGCTGCTATTGATGC
	vtc4-qPCR-R	AGCTGCTGTAGTTTCTTCACC
GalDH	galdh-qPCR-F	GCTACAGTCAGGGAGGCATT
	galdh-qPCR-R	GCTGAAAAGTCGAAACCCTCC
GLDH	gldh-qPCR-F	GAGGAGGTCTGTCGGTCATT
	gldh-qPCR-R	CATACTTCCTGAACTGTGCCTC
TDH3	tdh3-qPCR-F	TCTACCACCTCTCCAGTCCTTGTGGG
	tdh3-qPCR-R	ACCGCCCCAATGTTCGTCATGG

Table S3. The genes synthesized in this study

Genes	Source	Sequence (5'-3')
gme	*Arabidopsis thaliana*	ATGGGTACTACAAATGGTACTGATTACGGTGCTTACACATACAAGGAATTGGAAAGAGAACAATACTGGCC<br>ATCTGAAAATTTGAAAATTTCAATCACTGGTGCTGGTGGTTTTATTGCTTCTCATATCGCAAGAAGATTGAA<br>GCATGAAGGTCATTACGTTATCGCATCAGATTGGAAGAAAAATGAACATATGACTGAAGATATGTTCTGTGA<br>TGAATTTCATTTGGTTGATTTGAGAGTTATGGAAAACTGTTTGAAGGTTACAGAAGGTGTTGATCATGTTTT<br>TAATTTGGCTGCTGATATGGGTGGTATGGGTTTTATTCAATCTAATCATTCAGTTATTATGTACAACAACACA<br>ATGATTTCTTTTAATATGATCGAAGCTGCAAGAATTAATGGTATTAAAAGATTTTTCTATGCTTCTTCAGCATG<br>TATCTATCCAGAGTTTAAACAATTGGAAACTACAAATGTTTCTTTAAAAGAATCAGATGCTTGGCCAGCAGA<br>ACCACAAGATGCTTATGGTTTGGAAAAATTGGCAACTGAAGAATTGTGTAAGCATTACAATAAGGATTTCG<br>GTATCGAATGTAGAATCGGTAGATTCCATAATATCTATGGTCCATTTGGTACATGGAAAGGTGGTAGAGAAA<br>AAGCTCCAGCTGCATTTTGTAGAAAAGCACAAACTTCTACAGATAGATTTGAAATGTGGGGTGACGGTTTG<br>CAAACTAGATCCTTTACTTTTATTGATGAATGTGTTGAAGGTGTTTTGAGATTAACTAAGTCTGATTTCAGAG<br>AACCAGTTAACATCGGTTCTGATGAAATGGTTTCAATGAACGAAATGGCTGAAATGGTTTTGTCTTTTGAA<br>GAAAAGAAATTGCCAATCCATCATATTCCAGGTCCAGAAGGTGTTAGAGGTAGAAATTCAGATAACAATTTG<br>ATTAAAGAAAAATTGGGTTGGGCTCCAAATATGAGATTGAAAGAAGGTTTAAGAATCACTTACTTCTGGATC<br>AAGGAACAAATTGAAAAGGAAAAGGCTAAGGGTTCTGATGTTTCATTATACGGTTCTTCAAAAGTTGTTGG<br>TACACAAGCACCAGTTCAATTGGGTTCATTAAGAGCTGCTGATGGTAAAGAATAA
vtc2	*Arabidopsis thaliana*	ATGTTGAAGATTAAAAGAGTTCCAACAGTTGTTTCTAACTACCAAAAGGATGATGGTGCTGAAGATCCAGT<br>TGGTTGTGGTAGAAATTGTTTAGGTGCTTGTTGTTTGAATGGTGCAAGATTACCATTGTACGCTTGTAAGA<br>ATTTGGTTAAGTCTGGTGAAAAATTGGTTATTTCACATGAAGCAATTGAACCACCAGTTGCATTTTTGGAAT<br>CTTTAGTTTTGGGTGAATGGGAAGATAGATTCCAAAGAGGTTTGTTTAGATACGATGTTACAGCATGTGAAA<br>CTAAAGTTATTCCTGGTAAATACGGTTTCGTTGCTCAATTGAACGAAGGTAGACATTTGAAGAAAAGACCA<br>ACAGAGTTTAGAGTTGATAAGGTTTTACAATCTTTTGATGGTTCAAAGTTTAATTTTACTAAGGTTGGTCAA<br>GAAGAATTGTTGTTCCAATTTGAAGCTGGTGAAGATGCACAAGTTCAATTTTTCCCATGTATGCCAATTGAT<br>CCAGAAAATTCTCCATCAGTTGTTGCAATTAATGTTTCTCCAATCGAATACGGTCATGTTTTGTTGATCCCA<br>AGAGTTTTGGATTGTTTGCCACAAAGAATCGATCATAAATCTTTGTTGTTGGCTGTTCATATGGCTGCTGAA<br>GCTGCAAACCCATACTTCAGATTAGGTTACAACTCATTGGGTGCTTTCGCAACAATTAATCATTTGCATTTC<br>CAAGCATATTACTTAGCAATGCCATTTCCATTGGAAAAGGCTCCAACTAAGAAAATTACTACAACTGTTTCT<br>GGTGTTAAAATTTCTGAATTATTGTCATACCCAGTTAGATCATTATTGTTTGAAGGTGGTTCTTCAATGCAAG<br>AATTATCTGATACTGTTTCAGATTGTTGTGTTTGTTTACAAAACAACAACATCCCTTTTAATATTTTGATTTCT<br>GATTGTGGTAGACAAATTTTCTTGATGCCACAATGTTATGCTGAAAAACAAGCATTAGGTGAAGTTTCACCA<br>GAAGTTTTGGAAACACAAGTTAATCCAGCAGTTTGGGAAATTTCTGGTCATATGGTTTTGAAGAGAAAGGA<br>AGATTACGAAGGTGCTTCAGAAGATAATGCATGGAGATTATTGGCTGAAGCATCTTTGTCAGAAGAAAGAT<br>TCAAAGAAGTTACTGCTTTAGCATTTGAAGCTATCGGTTGTTCTAACCAAGAAGAAGATTTGGAAGGTACA<br>ATCGTTCATCAACAAAATTCTTCTGGTAACGTTAACCAAAAATCAAATAGAACTCATGGTGGTCCAATTACA<br>AATGGTACTGCTGCTGAATGTTTAGTTTTGCAATAA

Table S3（continue）

vtc4	*Arabidopsis thaliana*	ATGGCAGATAATGATCAATTTTTGGCTGCTGCTATTGATGCAGCTAAGAAAGCTGGTCAAATCATCAGAAA<br>GGGTTTCTACGAAACTAAACATGTTGAACATAAAGGTCAAGTTGATTTGGTTACTGAAACAGATAAAGGTT<br>GTGAAGAATTAGTTTTTAATCATTTGAAGCAATTGTTTCCAAACCATAAGTTTATTGGTGAAGAAACTACAG<br>CAGCTTTTGGTGTTACTGAATTGACAGATGAACCAACATGGATTGTTGATCCATTAGATGGTACTACAAACT<br>TCGTTCATGGTTTCCCATTCGTTTGTGTTTCTATCGGTTTAACTATCGGTAAAGTTCCAGTTGTTGGTGTTG<br>TTTACAACCCAATCATGGAAGAATTGTTTACAGGTGTTCAGGGTAAAGGTGCATTTTTAAACGGTAAAAGA<br>ATTAAAGTTTCTGCTCAATCAGAATTGTTGACTGCATTGTTGGTTACTGAAGCTGGTACAAAAAGAGATAAG<br>GCTACTTTGGATGATACTACAAACAGAATTAATTCTTTGTTGACAAAAGTTAGATCATTAAGAATGTCTGGTT<br>CATGTGCTTTGGATTTGTGTGGTGTTGCTTGTGGTAGAGTTGATATTTTCTATGAATTGGGTTTTGGTGGTC<br>CATGGGATATTGCAGCTGGTATTGTTATTGTTAAAGAAGCTGGTGGTTTGATCTTCGATCCATCTGGTAAAG<br>ATTTGGATATTACTTCACAAAGAATTGCAGCTTCTAACGCATCATTGAAGGAATTATTCGCAGAAGCATTGA<br>GATTAACAGGTGCTTAA
galdh	*Arabidopsis thaliana*	ATGACAAAGATTGAATTAAGGGCTTTGGGTAACACTGGATTGAAAGTCTCAGCTGTCGGATTTGGTGCATC<br>ACCATTGGGTTCAGTCTTTGGACCTGTCGCAGAGGACGACGCAGTCGCTACAGTCAGGGAGGCATTCAG<br>GTTGGGTATTAACTTTTTCGATACTTCACCTTACTACGGAGGTACATTGTCAGAAAAGATGTTGGGTAAGG<br>GATTGAAGGCTTTGCAGGTCCCTAGGTCAGACTACATTGTCGCAACAAAGTGCGGAAGATACAAGGAGG<br>GTTTCGACTTTTCAGCAGAGAGGGTCAGGAAGTCTATTGACGAGTCATTAGAGAGGTTGCAATTAGACTAT<br>GTTGATATTTTGCACTGCCATGACATTGAGTTCGGTTCATTGGATCAGATTGTTTCTGAAACAATTCCTGCT<br>TTGCAGAAGTTGAAGCAGGAGGGAAAGACAAGGTTTATTGGTATTACAGGTTTGCCATTAGACATTTTCAC<br>TTACGTCTTGGACAGGGTCCCACCTGGTACAGTCGACGTCATTTTGTCATACTGCCACTACGGAGTCAAC<br>GACTCAACTTTGTTGGACTTGTTGCCTTACTTAAAGTCAAAGGGTGTCGGAGTCATTTCAGCTTCTCCATT<br>GGCTATGGGATTGTTAACAGAGCAGGGTCCACCAGAGTGGCACCCAGCATCACCTGAGTTGAAGTCAGC<br>TTCTAAGGCAGCTGTCGCTCACTGCAAGTCTAAGGGTAAGAAAATTACTAAGTTGGCATTGCAGTACTCTT<br>TGGCAAATAAAGAGATTTCTTCAGTCTTGGTCGGTATGTCTTCAGTCTCTCAGGTCGAAGAGAACGTCGC<br>AGCTGTCACAGAGTTGGAGTCTTTGGGTATGGACCAAGAGACTTTGTCTGAGGTCGAGGCTATTTTGGA<br>GCCAGTCAAGAACTTGACTTGGCCATCTGGTATTCACCAGAACTAA

Table S3（continue）

gldh	*Arabidopsis thaliana*	ATGTTGAGATCTTTGTTGTTGAGGAGGTCTGTCGGTCATTCATTGGGAACTTTATCACCTTCTTCATCTACT<br>ATTAGGTCTTCTTTCTCTCCTCACAGGACATTGTGCACTACAGGACAGACTTTAACTCCACCTCCACCTCC<br>ACCACCAAGACCACCACCACCTCCTCCAGCAACAGCTTCTGAGGCACAGTTCAGGAAGTATGCTGGTTA<br>CGCAGCATTGGCTATTTTCTCAGGAGTCGCAACATACTTTTCTTTCCCTTTTCCAGAAAACGCAAAACACA<br>AGAAAGCACAGATTTTCAGGTACGCACCATTGCCTGAGGATTTGCACACTGTCTCAAACTGGTCTGGAAC<br>ACACGAGGTCCAGACTAGGAACTTCAACCAGCCTGAGAACTTGGCAGACTTGGAGGCATTGGTCAAAGA<br>GTCACACGAGAAGAAGTTAAGAATAAGACCAGTTGGTTCAGGTTTGTCACCAAACGGTATTGGATTGTCA<br>AGGTCTGGTATGGTTAACTTAGCTTTAATGGACAAGGTCTTGGAGGTCGACAAAGAGAAGAAGAGGGTCA<br>CTGTCCAGGCTGGTATTAGGGTTCAGCAGTTGGTCGACGCAATTAAGGACTACGGATTGACTTTGCAAAA<br>CTTCGCATCTATTAGGGAGCAACAAATTGGTGGTATAATACAGGTCGGTGCTCACGGTACAGGAGCTAGG<br>TTGCCTCCTATTGACGAACAGGTTATTTCAATGAAGTTAGTCACACCAGCTAAAGGAACTATAGAGTTATCA<br>AGGGAGAAGGACCCAGAGTTGTTCCACTTGGCAAGGTGCGGATTGGGTGGATTGGGAGTTGTCGCAGA<br>GGTCACTTTGCAGTGCGTCGCAAGGCATGAGTTAGTTGAGCACACATATGTCTCAAACTTGCAAGAGATA<br>AAGAAGAACCATAAGAAGTTGTTATCAGCAAATAAACACGTCAAATATTTGTACATACCTTATACAGACACA<br>GTCGTCGTCGTCACATGCAACCCTGTCTCAAAGTGGTCTGGACCTCCAAAGGACAAGCCAAAGTACACA<br>ACAGACGAGGCTGTCCAGCACGTTAGGGACTTGTACAGGGAGTCTATAGTCAAATACAGAGTCCAGGAC<br>TCTGGAAAGAAGTCTCCAGACTCTTCTGAGCCTGACATTCAGGAGTTATCTTTTACTGAATTGAGAGATAA<br>GTTGTTGGCATTGGATCCTTTGAATGACGTTCACGTTGCAAAAGTCAACCAGGCAGAGGCTGAGTTCTGG<br>AAGAAGTCAGAGGGTTACAGGGTCGGATGGTCAGACGAGATTTTGGGTTTCGATTGCGGAGGACAGCAG<br>TGGGTTTCTGAGTCTTGCTTCCCAGCTGGAACTTTGGCTAATCCATCTATGAAGGACTTGGAGTACATTGA<br>AGAGTTGAAGAAATTGATAGAGAAAGAAGCAATTCCTGCTCCAGCTCCTATAGAGCAGAGATGGACTGCT<br>AGGTCTAAATCTCCAATTTCACCAGCATTCTCAACTTCTGAGGACGACATTTTCTCTTGGGTTGGTATTATA<br>ATGTATTTGCCAACAGCTGATCCAAGACAGAGGAAGGATATTACTGACGAGTTTTTCACTACAGGCACTT<br>GACTCAGAAGCAGTTGTGGGACCAGTTCTCTGCATACGAGCACTGGGCTAAGATAGAGATACCTAAGGAT<br>AAGGAGGAATTGGAGGCTTTGCAGGCTAGGATAAGGAAGAGGTTCCCAGTCGACGCATACAACAAGGCT<br>AGAAGGGAGTTGGATCCAAACAGGATTTTGTCAAACAATATGGTCGAAAAATTGTTCCCAGTCTCTACTAC<br>TGCTTAA

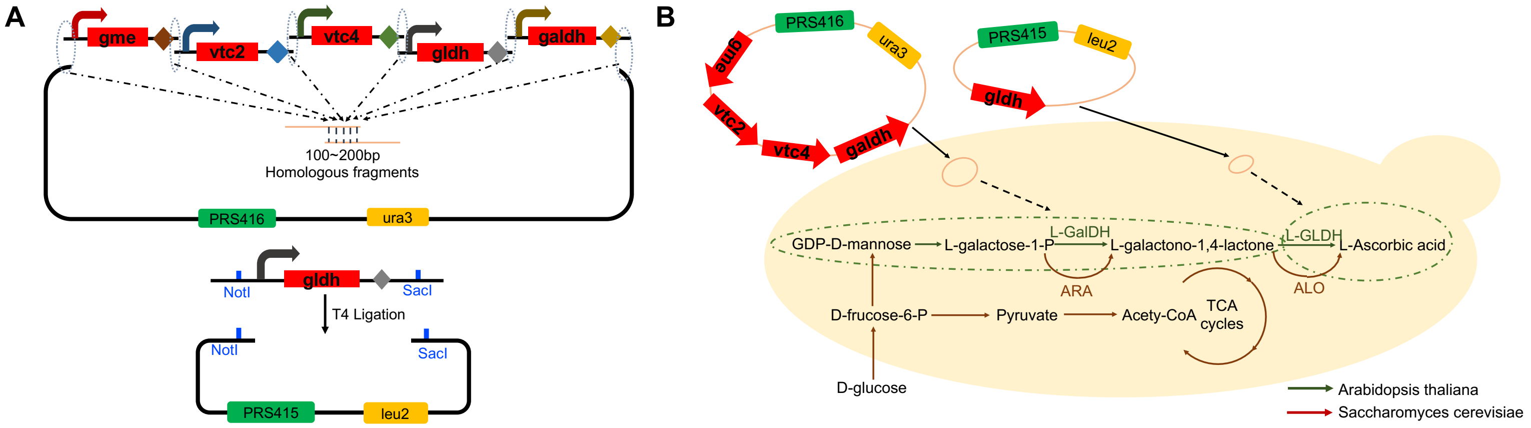

Fig. S1. The construction of YLAA. (A) The vector-construction used in YLAA. (B) The stain-construction used in YLAA.

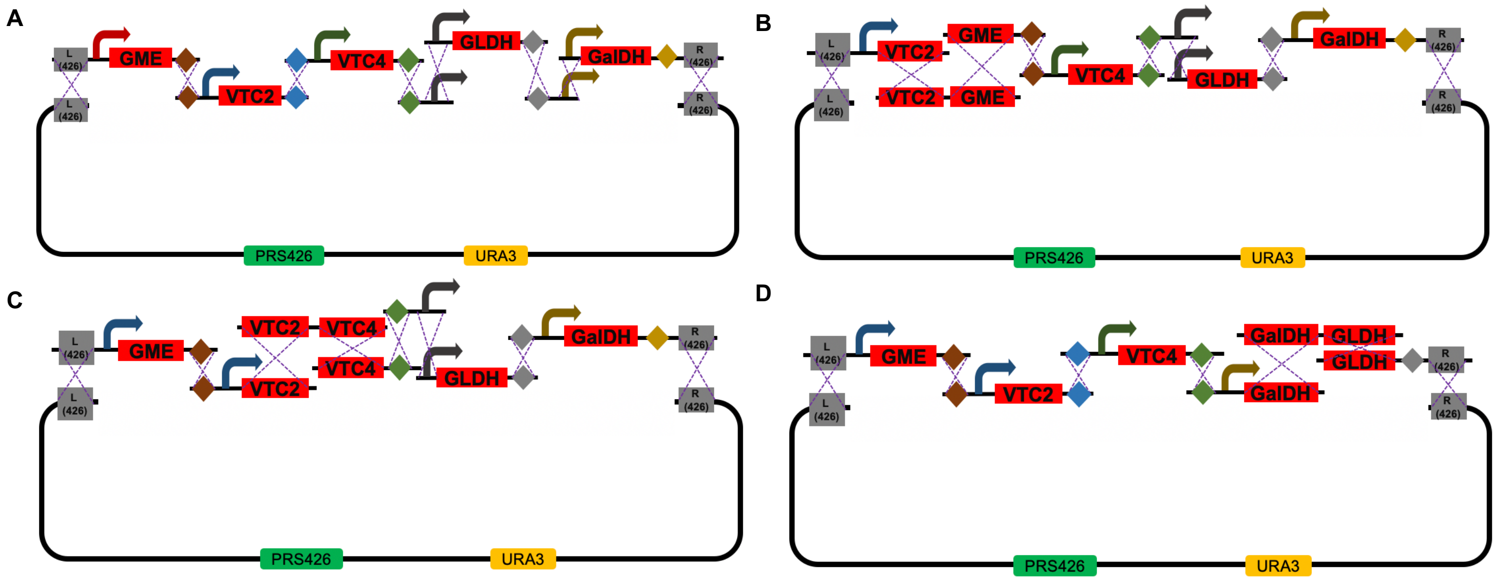

Fig. S2. The vector construction of strains with expressing exogenous modules and fusion-protein. (A) The vector construction of the exogenous-module-overexpression strain. (B) The vector construction of the strain with GME and GGP fusion expression, (C) GGP and GPP fusion expression and (D) L-GalDH and GLDH fusion expression.

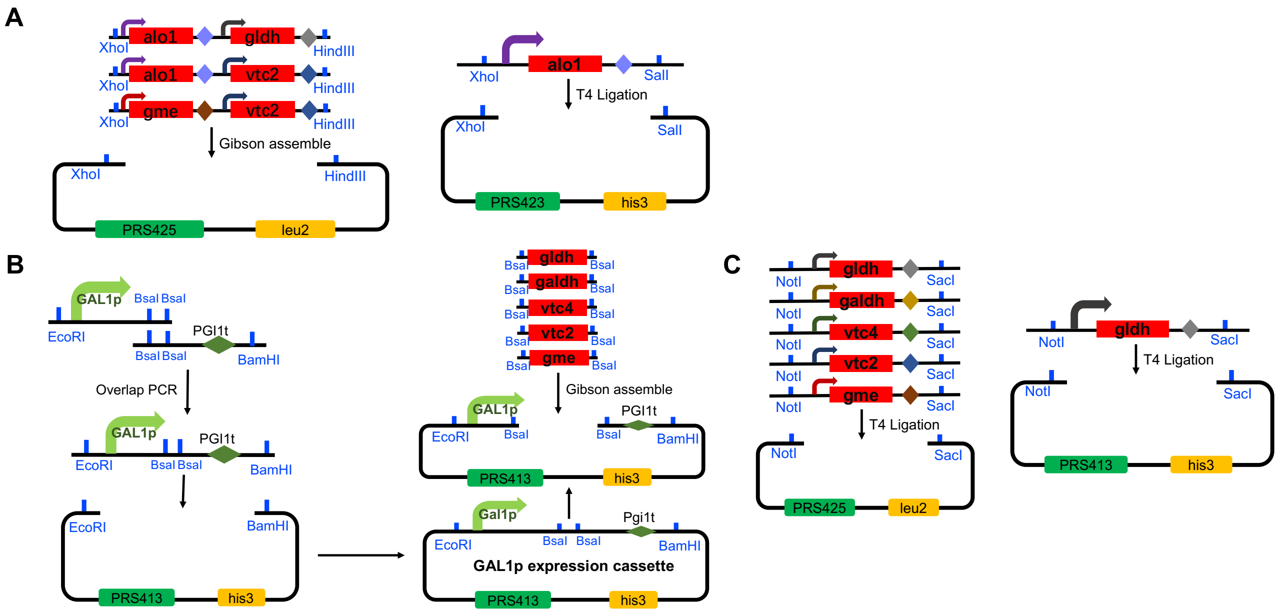

Fig. S3. The vector construction of the strains with overexpressing the rate-limiting genes. (A) The vector constructions of the strains with overexpressing different combinations of the possible rate-limiting genes. (B) The Gal1p-overexpression vector constructions. (C) The multicopy-overexpression vector constructions.

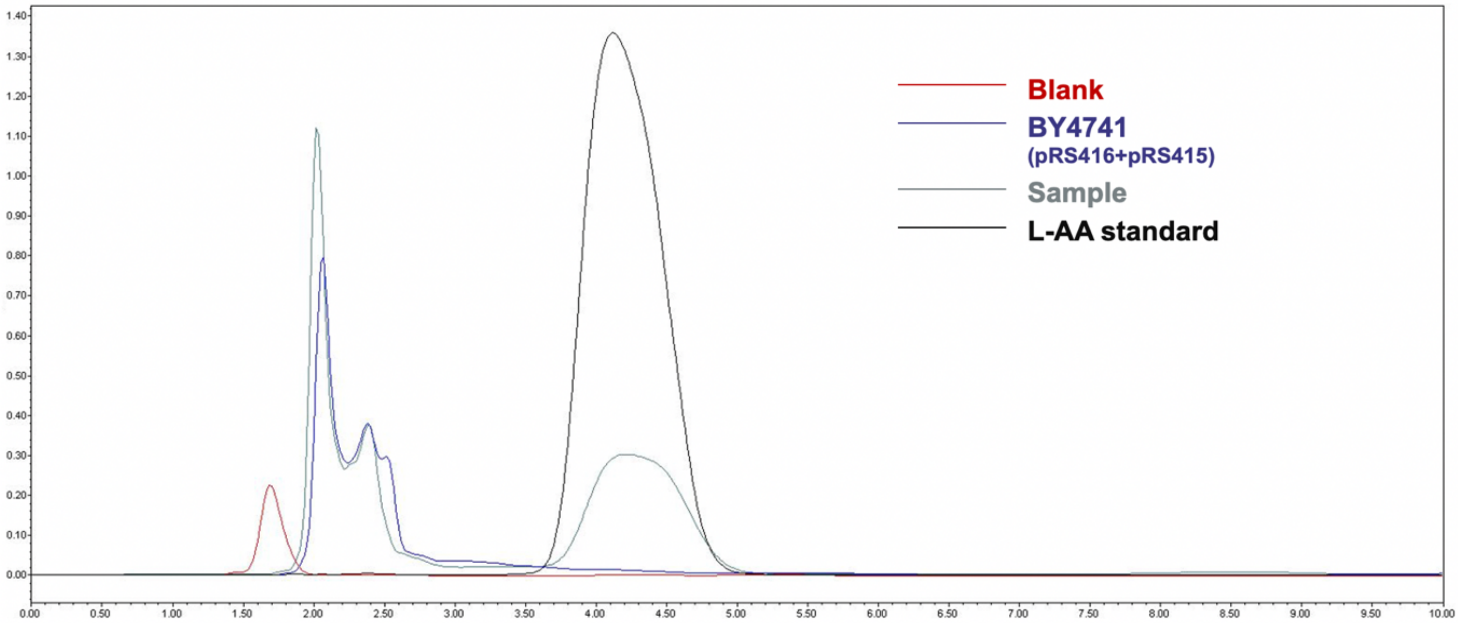

Fig.S4. Comparison figure of HPLC. Blank (Red line), BY4741(pRS416+pRS415) (Blue line), Sample (Gray line), L-AA standard (Black line).
